# Assessing the quality of life among children with cochlear implants in Saudi Arabia

**DOI:** 10.1371/journal.pone.0343237

**Published:** 2026-02-20

**Authors:** Faisl Alqraini

**Affiliations:** Department of Special Education, Prince Sattam bin Abdulaziz University, Al-Kharj, Saudi Arabia; LSU Health Shreveport, UNITED STATES OF AMERICA

## Abstract

Cochlear implantation has emerged as a transformative intervention for children with severe-to-profound hearing loss, improving not only auditory and linguistic abilities but also broader aspects of quality of life. This study examined school readiness in toddlers and preschool-aged children with cochlear implants in Saudi Arabia and explored the role of psychosocial factors—self-reliance, well-being and happiness, and social relationships—in predicting readiness outcomes. Using a cross-sectional correlational survey design, data were collected from 155 parents via an online questionnaire distributed in social media groups for families of children with hearing loss. Data were collected using an online parent-reported questionnaire disseminated through established WhatsApp and Telegram groups for families of children with hearing loss in Saudi Arabia. A non-probability convenience sampling technique was employed to recruit participants. Descriptive statistics indicated that most children were perceived as keeping pace with peers in educational settings, although parental concerns about future school placement were common. Pearson correlations revealed moderate positive associations between each psychosocial factor and school readiness, while multiple regression analysis identified social relationships as the only significant predictor when all factors were considered together. The findings underscore the importance of fostering strong peer and teacher relationships in early intervention programs, alongside promoting self-reliance and emotional well-being. These results have implications for educators, policymakers, and rehabilitation specialists seeking to enhance the educational readiness and quality-of-life outcomes of children with cochlear implants in Saudi Arabia.

## Introduction

Over the past two decades, cochlear implantation has emerged as a transformative intervention for children with severe-to-profound hearing loss, significantly improving their auditory perception, speech development, and overall quality of life [1]. In Saudi Arabia, advances in early detection, surgical techniques, and rehabilitation services have contributed to notable gains in functional outcomes for children with cochlear implants (CIs), particularly in their social relationships, emotional well-being, self-reliance, and academic performance [2]. By the end of 2020, approximately 5,800 children in Saudi Arabia had received cochlear implants [2]. These improvements, however, vary widely among individuals, highlighting the need to identify factors that contribute to optimal developmental trajectories.

One critical developmental milestone for children with CIs is school readiness, which refers to a child’s preparedness to engage successfully in formal education settings. School readiness is a multidimensional construct encompassing cognitive, socio-emotional, behavioral, and physical competencies that enable children to adapt to and thrive in structured learning environments [3]. While language development is a core component of readiness, non-linguistic psychosocial factors such as self-reliance, well-being, and social relationships also play essential roles in determining a child’s ability to cope with the academic and social demands of school [4,5].

Children with CIs may experience unique challenges in achieving school readiness due to residual hearing difficulties, delayed language acquisition, and limitations in incidental learning [6]. These challenges can affect not only their cognitive preparedness but also their social integration, self-confidence, and emotional adjustment [7]. In contexts such as Saudi Arabia, where educational opportunities for children with hearing loss have expanded yet continue to evolve, understanding the psychosocial determinants of school readiness is critical for developing targeted support strategies that address both academic and non-academic needs.

Psychosocial factors including self-reliance, well-being, and social relationships—are increasingly recognized as integral to educational success in children with hearing loss [8]. Self-reliance, defined as a child’s ability to function independently and persist in tasks, supports problem-solving skills and adaptability in classroom environments [9]. Well-being and happiness reflect positive emotional states that facilitate engagement, resilience, and sustained learning motivation [10]. Social relationships, encompassing peer acceptance, cooperative behaviors, and positive teacher-student interactions, contribute to a sense of belonging and can mitigate the effects of communication barriers [11].

Previous studies have documented that strong psychosocial functioning is associated with improved academic outcomes among children with CIs, even after accounting for language ability [12]. However, much of this evidence derives from Western contexts, such as North America, Western Europe, and Australia, and little is known about how these relationships manifest in Middle Eastern settings, particularly within Saudi Arabia’s cultural and educational landscape. Given the socio-cultural emphasis on family involvement and community integration in Saudi society, psychosocial dynamics may have distinct implications for school readiness in this population [2].

The present study addresses this gap by examining the school readiness of toddlers and preschoolers with cochlear implants in Saudi Arabia and exploring the influence of three psychosocial factors self-reliance, well-being, and happiness, and social relationships—on readiness outcomes. By identifying the most salient predictors, the study aims to inform intervention strategies that enhance the preparedness of children with CIs for formal schooling, ultimately supporting their long-term educational and social success.

Accordingly, the study was guided by the following research questions:

1. What is the level of school readiness among children with cochlear implants in Saudi Arabia?
2. Do psychosocial factors, including self-reliance, well-being and happiness, and social relationships, influence the school readiness of children with cochlear implants in Saudi Arabia?

## Method

### Research design

This study employed a cross-sectional correlational survey design utilizing an online parent-reported questionnaire [13]. The instrument comprised demographic items and validated scales assessing psychosocial factors and school readiness. Content validity was evaluated by experts in special education, audiology, and early childhood development, while construct validity was assessed using principal components analysis. Data were collected electronically between 12 July and 14 September 2024 through a non-probability convenience sampling approach via established WhatsApp and Telegram groups for families of children with hearing loss in Saudi Arabia.

### Participants

Participants were parents or primary caregivers of children with cochlear implants residing in Saudi Arabia. Recruitment was conducted online through targeted outreach in WhatsApp and Telegram groups where families of children with hearing loss share information and experiences. An invitation message containing a link to the electronic questionnaire and information about the study was distributed to 524 families. Participants were identified through widely utilized Saudi parent-support communities on these platforms, which served as the primary recruitment channels for disseminating the survey invitation. Of these, 155 families completed the survey, yielding a response rate of 29.6%.

The children represented in the sample ranged in age from 1 to 5 years (M = 3.4 years), with 55% identified as female. Notably, 22% of participating families reported having more than one child with a cochlear implant, indicating multiple experiences with the intervention. Table 1 summarizes the sociodemographic characteristics of the participating families.

**Table 1:** Sociodemographic Characteristics of the Participants.

Variable	Value
Number of responding families	155
Response rate	29.6%
Child age range	1–5 years (M = 3.4)
Child gender	55% female (n ≈ 85), 45% male (n ≈ 70)
Families with more than one child with a cochlear implant	22% (n ≈ 34 families)

### Sampling strategy and sample size determination

A non-probability convenience sampling strategy was used, consistent with social media–based recruitment methods in similar research contexts [14,15]. To determine the required sample size, an a priori power analysis was conducted using GPower 3.1 for a linear multiple regression model with three predictors representing the three psychosocial factors examined in this study [16]. A small-to-medium anticipated effect size (f² = 0.10) was selected based on recommendations for behavioral and psychosocial research, with the alpha level set at 0.05 and the desired statistical power at 0.90. According to these parameters, GPower indicated that a minimum of 146 participants was required. The achieved sample of 155 participants therefore met and exceeded the recommended threshold for adequate statistical power.

### Measures

#### Demographic characteristics.

Section 1 of the questionnaire collected demographic data, including the child’s gender, age, and family background, using single-item, multiple-choice questions.

#### Psychosocial factors and school readiness.

Section 2 measured three psychosocial constructs self-reliance, well-being and happiness, and social relationships alongside school readiness. All items in this section used a five-point Likert scale (1 = strongly disagree to 5 = strongly agree), which is widely used to assess attitudes, perceptions, and self-reported behaviors in quantitative research [17].

The items used in this study were adapted from the Children with Cochlear Implants: Parental Perspectives (CCIPP) instrument developed by Archbold and colleagues [18]. The CCIPP is widely recognized as one of the most reliable and extensively used tools for assessing quality-of-life outcomes in children with cochlear implants, and it has been recommended for both clinical evaluation and research purposes [18,19]. Its adaptability has been demonstrated across multiple cultural contexts, including Brazil [20], Finland [21], China [22], the United States [23], Lithuania [24], and Armenia [25]. Collectively, this evidence supports the CCIPP as a robust and culturally adaptable measure for capturing parental perceptions of children’s post-implantation functioning [19,26].

Content validity was assessed by a panel of five experts in audiology, special education, and early childhood development. Each item was evaluated for relevance, clarity, and cultural appropriateness. The overall Content Validity Index (CVI) was 0.89, indicating strong agreement regarding item relevance. The Content Validity Ratio (CVR) was 0.86, exceeding the minimum threshold recommended by Lawshe for expert consensus. These values demonstrate that the instrument possesses acceptable content validity for use within the Saudi context.

*A detailed overview of the survey instrument, including the variables, number of items, and measurement scales, is presented in* Table 2.

**Table 2:** Survey Instrument Description.

Section	Variables	Number of Items	Measurement
**Section 1: Demographics**	Gender, Age, Family Background	Single-item variables	Multiple-choice
**Section 2: Psychosocial constructs and school readiness**	Self-Reliance	5	5-point Likert scale
	Well-being and Happiness	8	
	Social Relationships	13	
	School Readiness	11	

### Instrument validity and reliability

#### Content validity.

Content validity was established through expert review. Subject-matter experts in special education, early childhood development, and audiology evaluated the questionnaire to ensure that all items were relevant, clear, and representative of their respective constructs [27]. Feedback from the reviewers informed revisions to improve clarity and comprehensiveness.

#### Construct validity.

Construct validity was examined using principal components analysis (PCA). Bartlett’s test of sphericity and the Kaiser–Meyer–Olkin (KMO) measure confirmed the suitability of the data for factor analysis. Each construct demonstrated unidimensionality, with factor loadings for retained items ranging from 0.567 to 0.807. Items with loadings below 0.30 were removed to strengthen construct coherence.

#### Reliability.

Internal consistency reliability was assessed using Cronbach’s alpha [28]. Acceptable reliability was observed for all constructs: self-reliance (α = 0.699), well-being and Happiness (α = 0.703), social relationships (α = 0.807), and school readiness (α = 0.650). Items that reduced alpha scores were removed during the scale refinement process.

Cronbach’s alpha values for each construct, along with the number of items retained after refinement, are reported in Table 3.

**Table 3:** Internal Consistency Reliability.

Construct	Cronbach’s Alpha	Initial Items	Final Items
**Self-Reliance**	0.699	4	4
**Well-being and Happiness**	0.703	7	7
**Social Relationships**	0.807	13	13
**School Readiness**	0.65	4	4

### Data collection

The questionnaire was distributed via Google Forms to 524 families, of which 155 responded. Participation was voluntary, and only those who provided informed consent were included in the study. The consent form was embedded at the start of the online survey, and participants were required to indicate agreement before they could proceed. This ensured that written informed consent in electronic form was documented digitally. Data collection was completed in 33 days, between 12 July 2024 and 14 September 2024.

### Ethical considerations

Ethical approval for the study was obtained from Prince Sattam bin Abdulaziz University, Deanship of Scientific Research (No: 305/2024). To ensure confidentiality and anonymity, no identifying information was collected, and all responses were stored without any personal identifiers. Participants accessed the questionnaire through a secure and encrypted survey link, and data were stored on password-protected servers accessible only to the research team. Although recruitment occurred via social media platforms, no private user information was accessed; group administrators only distributed the survey link. All data handling procedures adhered to institutional ethical guidelines and complied with best practices for digital data security in online research. Participants were assured of confidentiality, anonymity, and voluntary participation, and informed consent was obtained electronically prior to completing the survey.

### Data analysis

Data analysis was conducted using IBM SPSS Statistics version 27. Descriptive statistics, including frequency distributions and percentages, were used to summarize demographic characteristics and assess the level of school readiness in the sample. The Shapiro–Wilk test was used to examine the normality of the dependent variable (school readiness), confirming suitability for parametric analysis.

Pearson’s correlation coefficients were calculated to examine the relationships among the three psychosocial constructs and school readiness. Multiple linear regression analysis was then conducted to assess the predictive power of self-reliance, well-being and happiness, and social relationships on school readiness. Statistical significance was set at p < .05 for all analyses.

## Results

### Descriptive analysis of school readiness

The first research question examined the level of school readiness among children with cochlear implants in Saudi Arabia. All items met the minimum thresholds for reliability and conceptual relevance; therefore, no items were removed during the refinement process. The majority of respondents (72.9%) agreed or strongly agreed that their child was keeping up well with peers of the same age at school. Conversely, 41.3% disagreed or strongly disagreed that their child was unable to cope with mainstream schooling. Regarding satisfaction with current educational placement, 40.0% disagreed or strongly disagreed with the statement “I am not happy with his educational placement at present.” However, concerns about future school placement remained prevalent, with 69.7% agreeing or strongly agreeing with the statement “I am concerned about his future school placement.”

Therefore, the findings provide a clear answer to Research Question 1 by showing that children with cochlear implants demonstrate generally positive levels of school readiness, although concerns about future school placement remain relatively common Table 4.

**Table 4:** Frequencies for School Readiness Items.

Item	Strongly Disagree (%)	Disagree (%)	Neither Agree nor Disagree (%)	Agree (%)	Strongly Agree (%)
**Keeps up well with peers**	1.9	9.7	15.5	39.4	33.5
**Unable to cope with mainstream schooling**	12.3	29	29	19.4	10.3
**Not happy with current educational placement**	12.9	27.1	28.4	15.5	16.1
**Concerned about future school placement**	2.6	11.6	16.1	24.5	45.2

### Correlation analysis

To assess the potential impact of multicollinearity among the psychosocial predictors, Variance Inflation Factor (VIF) values were examined. All VIF values ranged from 1.41 to 1.65, well below the commonly accepted threshold of 5.0, indicating no evidence of problematic multicollinearity. These results suggest that the moderate correlations observed among predictors did not distort the regression estimates.

Pearson correlation coefficients were calculated to examine the associations between psychosocial factors and school readiness. Adjusted odds ratios (AOR) could not be calculated because the study design and available dataset did not support multivariable logistic regression. Instead, unadjusted odds ratios are presented, which remain appropriate and interpretable within the context of an exploratory cross-sectional design Table 5.

**Table 5:** Pearson Correlation Coefficients Between Psychosocial Factors and School Readiness.

Variable	Self-Reliance	Well-being & Happiness	Social Relationships	School Readiness
**Self-Reliance**	1	.603**	.621**	.411**
**Well-being & Happiness**	.603**	1	.685**	.427**
**Social Relationships**	.621**	.685**	1	.557**
**School Readiness**	.411**	.427**	.557**	1

Note: p < .001 (two-tailed).

All three psychosocial variables were positively and significantly correlated with school readiness. The correlations were of moderate strength, suggesting that higher levels of self-reliance, well-being and happiness, and social relationships were each associated with greater readiness for school.

These results directly address Research Question 2, demonstrating that all three psychosocial factors self-reliance, well-being and happiness, and social relationships are positively associated with school readiness.

### Multiple regression analysis

A standard multiple regression was conducted to determine the extent to which the three psychosocial variables collectively predicted school readiness Table 6.

**Table 6:** Multiple Regression Analysis Predicting School Readiness from Psychosocial Factors.

Predictor	B	SE B	β	t	p
**Constant**	−0.209	0.405	—	−0.516	0.607
**Self-Reliance**	0.096	0.095	0.091	1.011	0.314
**Well-being & Happiness**	0.072	0.125	0.056	0.573	0.568
**Social Relationships**	0.651	0.139	0.462	4.678	<.001

Note. Results are based on a standard multiple linear regression predicting school readiness from the three psychosocial variables (self-reliance, well-being and happiness, and social relationships). Coefficients represent unstandardized (B) and standardized (β) effects. No logistic regression or odds ratios were calculated.

The overall model was statistically significant, F(3, 151) = 23.518, p < .001, explaining 30.5% of the variance in school readiness (adjusted R² = .305). Social relationships were the only statistically significant predictor when controlling for the other variables.

Accordingly, the findings answer Research Question 3 by showing that social relationships are the only statistically significant predictor of school readiness when controlling for self-reliance and well-being and happiness.

## Discussion

The present study investigated two central research questions: (1) What is the level of school readiness among toddlers and preschool-aged children with cochlear implants (CIs) in Saudi Arabia? and (2) To what extent do psychosocial factors self-reliance, well-being and happiness, and social relationships influence school readiness in this population? The findings provide empirical insights into the educational and psychosocial dimensions of cochlear implantation in early childhood within a Middle Eastern context, thereby extending existing literature that has largely been generated in Western settings.

### School readiness in children with cochlear implants

In relation to the first research question, descriptive analyses revealed that a substantial proportion of parents perceived their children with CIs as keeping pace with age-matched peers, suggesting that early implantation coupled with rehabilitative support can enable children to attain age-appropriate readiness for school entry. This is consistent with prior research indicating that auditory access provided through CIs, when combined with structured intervention, can facilitate age-level attainment in cognitive, linguistic, and socio-emotional domains [2,6].

Nevertheless, the finding that nearly 70% of parents expressed concern about their child’s future educational placement underscores the persistent challenges faced by this group. Such concerns likely reflect apprehension about the sustainability of initial readiness gains, particularly in mainstream educational environments where specialized supports may not be consistently available. This aligns with international evidence highlighting that while early gains are attainable, the maintenance of positive outcomes often depends on ongoing educational accommodations and inclusive pedagogical practices [7].

### Psychosocial factors and school readiness

The second research question addressed the predictive value of psychosocial constructs in explaining variability in school readiness. Correlational analyses indicated that self-reliance, well-being and happiness, and social relationships were each significantly and positively associated with readiness. This affirms the multidimensional nature of school readiness, which extends beyond cognitive and linguistic competencies to encompass socio-emotional capacities and interpersonal functioning [4,5].

However, regression analysis revealed that when considered simultaneously, only social relationships remained a statistically significant predictor. This finding suggests that while self-reliance and well-being may contribute to readiness, their effects could be mediated through the quality of a child’s social connections. In other words, social relationships may serve as the primary mechanism by which other psychosocial strengths translate into adaptive functioning in school contexts. This interpretation resonates with socioecological perspectives [29] and with empirical evidence indicating that peer acceptance, cooperative engagement, and positive teacher–child interactions are strong determinants of academic adjustment for children with hearing loss [12].

### Theoretical and contextual interpretation

From a theoretical standpoint, these findings reinforce the ecological systems model of development, which posits that the child’s immediate social environments particularly the mesosystem linking home and school are critical determinants of readiness. In the Saudi Arabian cultural context, where family cohesion and collective social engagement are emphasized, early peer integration and teacher rapport may exert even stronger influences on educational outcomes. Social connectedness not only facilitates engagement in classroom activities but also supports incidental learning, emotional regulation, and resilience, all of which are foundational to readiness.

In the Saudi Arabian context, cultural factors may play a significant role in shaping psychosocial development and school readiness among children with cochlear implants. Saudi society places strong emphasis on family involvement, social cohesion, and collective responsibility, which may influence children’s opportunities for social interaction and emotional development. Extended family networks and community support systems often provide substantial assistance, potentially enhancing social relationships and well-being. At the same time, cultural expectations regarding communication norms, early childhood behavior, and parental roles may influence how parents perceive and report their child’s readiness for school. Understanding these cultural dynamics is essential for interpreting psychosocial outcomes within this population and for designing interventions that align with local values and family practices [7].

Societal attitudes toward disability and cochlear implant use in Saudi Arabia may also influence the psychosocial development of children. Although awareness and acceptance of hearing technologies have increased, some families may still encounter social stigma or misunderstandings regarding communication differences and device use. Such perceptions may affect a child’s confidence, peer interactions, and emotional well-being, particularly in early educational settings. Conversely, in communities where cochlear implantation is viewed positively and supported by strong advocacy networks, children may experience greater social inclusion and encouragement. Recognizing the role of societal attitudes is therefore essential for interpreting psychosocial outcomes and for designing interventions that promote inclusive environments for children with cochlear implants [2].

The mediating potential of social relationships observed here aligns with quality-of-life frameworks in pediatric audiology, which advocate for outcome measures that incorporate social participation and integration alongside auditory and linguistic benchmarks. The evidence from this study supports the proposition that social participation is not merely a byproduct of successful implantation but may be a central driver of broader developmental success.

### Implications for policy and practice

These findings suggest a need to broaden early intervention and preschool readiness programs for children with CIs to explicitly target the development of social competence. Recommended strategies include:

- Embedding structured opportunities for peer collaboration and interaction within the preschool curriculum.
- Training teachers in inclusive practices that foster positive peer dynamics and address communication barriers.
- Creating school-based initiatives that integrate children with CIs into group learning experiences to strengthen both academic and social engagement.

Furthermore, although self-reliance and well-being were not unique predictors in the regression model, their moderate correlations with readiness suggest that they remain important developmental targets. These domains may be best supported through activities that simultaneously enhance social engagement such as group problem-solving tasks or cooperative play which can reinforce independence and emotional resilience within a socially meaningful context.

Parental engagement emerges as another critical factor. Facilitating active parent–school communication and providing families with strategies to promote social interaction outside the classroom such as participation in community-based groups and extracurricular activities can help sustain the social competencies that underpin readiness.

### Limitations and future directions

First, the use of a non-probability convenience sampling strategy limits the generalizability of the findings to all families of children with cochlear implants in Saudi Arabia. Because the sample consisted of parents who were active in online support groups and willing to participate in an electronic survey, it may not fully represent the broader population. Therefore, the findings should be interpreted with caution and regarded as exploratory rather than generalizable.

Second, the response rate of 29.6% may limit the representativeness of the sample. Families who chose to participate may differ systematically from those who did not, introducing the potential for non-response bias and affecting the extent to which the results reflect the perspectives of all families of children with cochlear implants.

Third, psychosocial variables were assessed solely through parental self-report, which may introduce perceptual and single-informant bias. Parents may unintentionally overestimate or underestimate their child’s behaviors or social functioning due to personal expectations or limited observation across settings. The absence of teacher, clinician, or observational data further limits the objectivity of these measures. Future studies should incorporate multi-informant assessments to enhance measurement accuracy.

Fourth, the study did not incorporate alternative data sources such as teacher evaluations, clinician reports, or direct classroom observations. Relying on a single informant limits triangulation and may not fully capture variations in children’s behavior across settings. Integrating multiple data sources would improve ecological validity.

Fifth, several key variables known to influence developmental outcomes such as severity of hearing loss, age at cochlear implantation, duration and quality of rehabilitation services, and socioeconomic status—were not included in the analyses. Their omission restricts the ability to isolate the unique contribution of the psychosocial predictors. Future studies should incorporate these covariates into more comprehensive and controlled models.

Sixth, the school readiness construct demonstrated a Cronbach’s alpha of 0.65, which is slightly below conventional thresholds for internal consistency. Although retained based on conceptual relevance and factor structure, this reduced reliability suggests the scale may not fully capture the multidimensional nature of school readiness. Further psychometric validation is warranted.

Seventh, some survey items showed weaker factor loadings during principal components analysis and were removed to strengthen construct validity. This refinement improved scale coherence but suggests the initial item pool may not have fully represented all dimensions of the constructs. Future work should expand item pools and conduct additional validation.

Eighth, the cross-sectional design limits causal inference and prevents determining whether psychosocial functioning influences school readiness, whether readiness influences psychosocial functioning, or whether the relationship is bidirectional. Longitudinal designs are needed to clarify directional pathways and developmental change over time.

Ninth, the study focused primarily on three psychosocial constructs self-reliance, well-being, and social relationships despite the broader range of factors known to shape school readiness, including cognitive abilities, language development, emotional regulation, resilience, and family dynamics. Incorporating these domains in future research would yield a more holistic understanding of readiness.

Tenth, the use of online recruitment via WhatsApp and Telegram may have introduced digital access bias. Families with greater technological access or engagement were more likely to respond, potentially underrepresenting households with limited digital resources and further constraining generalizability.

Eleventh, although multicollinearity diagnostics indicated acceptable VIF values, moderate intercorrelations among psychosocial constructs (e.g., r = 0.62 between social relationships and self-reliance) may reflect conceptual overlap that influences their relative predictive strengths. Structural equation modeling could help disentangle shared variance from unique contributions in future studies.

Twelfth, although social relationships emerged as the strongest predictor of school readiness, the study did not examine potential causal mechanisms or interaction effects among predictors. Constructs such as self-reliance or well-being may influence readiness indirectly through social connectedness. Mediation or moderation analyses would help clarify these pathways.

Finally, the findings reflect the cultural and educational context of families of children with cochlear implants in Saudi Arabia and may not be generalizable to other regions or populations. Variations in cultural norms, educational systems, and rehabilitation services across countries may shape psychosocial functioning and school readiness in different ways. Comparative cross-cultural research would help determine the extent to which these findings are context-specific or universally applicable.

## Conclusion

This study addressed its stated goals by documenting that young children with CIs in Saudi Arabia generally demonstrate positive school readiness outcomes and by identifying social relationships as the most salient psychosocial predictor of readiness. The findings underscore the necessity of integrating social competence development into early educational planning and intervention. By foregrounding the centrality of social relationships, the results provide a clear and actionable direction for educational and therapeutic strategies aimed at enhancing both readiness and long-term quality-of-life outcomes for children with cochlear implants.

## Supporting information

S1 Appendix The questionnaire used for data collection (English and Arabic versions). (PDF)

## References

[1] Alqraini F. Knowledge level of teachers of the d/deaf and hard of hearing to develop phonological awareness of students with cochlear implants. JETT. 2023;14(4). doi: 10.47750/jett.2023.14.04.024

[2] Alqraini FM. Quality of life of children with cochlear implants in Saudi Arabia. Res Dev Disabil. 2025;161:105007. doi: 10.1016/j.ridd.2025.105007 40215599

[3] Snow KL. Developing kindergarten readiness and other large-scale assessment systems: Necessary considerations in the assessment of young children. National Institute for Early Education Research. 2011.

[4] Britto PR, Lye SJ, Proulx K, Yousafzai AK, Matthews SG, Vaivada T, et al. Nurturing care: Promoting early childhood development. Lancet. 2017;389(10064):91–102. doi: 10.1016/S0140-6736(16)31390-3 27717615

[5] Hair E, Halle T, Terry-Humen E, Lavelle B, Calkins J. Children’s school readiness in the ECLS-K: Predictions to academic, health, and social outcomes in first grade. Early Childhood Research Quarterly. 2006;21(4):431–54. doi: 10.1016/j.ecresq.2006.09.005

[6] Niparko JK, Tobey EA, Thal DJ, Eisenberg LS, Wang N-Y, Quittner AL, et al. Spoken language development in children following cochlear implantation. JAMA. 2010;303(15):1498–506. doi: 10.1001/jama.2010.451 20407059 PMC3073449

[7] Hyde M, Punch R. The modes of communication used by children with cochlear implants and the role of sign in their lives. Am Ann Deaf. 2011;155(5):535–49. doi: 10.1353/aad.2011.0006 21449251

[8] Antia SD, Jones P, Luckner J, Kreimeyer KH, Reed S. Social outcomes of students who are deaf and hard of hearing in general education classrooms. Exceptional Children. 2011;77(4):489–504. doi: 10.1177/001440291107700407

[9] Denham SA, Bassett HH, Thayer SK, Mincic MS, Sirotkin YS, Zinsser K. Observing preschoolers’ social-emotional behavior: structure, foundations, and prediction of early school success. J Genet Psychol. 2012;173(3):246–78. doi: 10.1080/00221325.2011.597457 22919891

[10] Lyubomirsky S, King L, Diener E. The benefits of frequent positive affect: Does happiness lead to success?. Psychol Bull. 2005;131(6):803–55. doi: 10.1037/0033-2909.131.6.803 16351326

[11] Calderon R, Greenberg MT. Social and emotional development of deaf children: family, school, and program effects. The Oxford Handbook of Deaf Studies, Language, and Education, Volume 1, Second Edition. Oxford University Press. 2012. p. 188–99. doi: 10.1093/oxfordhb/9780199750986.013.0014

[12] Wiefferink CH, Rieffe C, Ketelaar L, Frijns JHM. Predicting social functioning in children with a cochlear implant and in normal-hearing children: The role of emotion regulation. Int J Pediatr Otorhinolaryngol. 2012;76(6):883–9. doi: 10.1016/j.ijporl.2012.02.065 22459035

[13] Wang X, Cheng Z. Cross-Sectional Studies: Strengths, Weaknesses, and Recommendations. Chest. 2020;158(1S):S65–71. doi: 10.1016/j.chest.2020.03.012 32658654

[14] Fricker RD. Sampling methods for web and E-mail surveys. The SAGE Handbook of Online Research Methods. SAGE Publications, Ltd. 2008. 195–216. doi: 10.4135/9780857020055.n11

[15] Ohei K, Chukwuere JE, Adom D, Chukwuere PC. Social media research: Sampling techniques, data collection, analysis, and discussion. A–Z of social media research methods. New York: Springer. 2022. p. 104–27.

[16] Faul F, Erdfelder E, Lang A-G, Buchner A. G*Power 3: A flexible statistical power analysis program for the social, behavioral, and biomedical sciences. Behav Res Methods. 2007;39(2):175–91. doi: 10.3758/bf03193146 17695343

[17] Batterton KA, Hale KN. The Likert scale what it is and how to use it. Phalanx. 2017;50:32–9.

[18] Archbold SM, Lutman ME, Gregory S, O’Neill C, Nikolopoulos TP. Parents and their deaf child: Their perceptions three years after cochlear implantation. Deafness & Education International. 2002;4(1):12–40. doi: 10.1179/146431502790560962

[19] Archbold T, Nunes M, Lutman U, Pretzlik S, Gregory S. Parental perspectives of children with cochlear implants: The validated questionnaire. Measuring the immeasurable? Proceedings of a conference on quality of life in deaf children. Oxford: Hughes Associates. 2003.

[20] Fortunato-Tavares T, Befi-Lopes D, Bento RF, Andrade CRF. Children with cochlear implants: Communication skills and quality of life. Braz J Otorhinolaryngol. 2012;78(1):15–25. doi: 10.1590/s1808-86942012000100003 22392233 PMC9443866

[21] Huttunen K, Rimmanen S, Vikman S, Virokannas N, Sorri M, Archbold S, et al. Parents’ views on the quality of life of their children 2-3 years after cochlear implantation. Int J Pediatr Otorhinolaryngol. 2009;73(12):1786–94. doi: 10.1016/j.ijporl.2009.09.038 19875180

[22] Zhao Y, Li Y, Zheng Z, Li J, Nie X, Jin X, et al. Health-related quality of life in mandarin-speaking children with cochlear implants. Ear Hear. 2019;40(3):605–14. doi: 10.1097/AUD.0000000000000633 30063476

[23] Kumar R, Warner-Czyz A, Silver CH, Loy B, Tobey E. American parent perspectives on quality of life in pediatric cochlear implant recipients. Ear Hear. 2015;36(2):269–78. doi: 10.1097/AUD.0000000000000108 25377531

[24] Byčkova J, Simonavičienė J, Mickevičienė V, Lesinskas E. Evaluation of quality of life after paediatric cochlear implantation. Acta Med Litu. 2018;25(3):173–84. doi: 10.6001/actamedica.v25i3.3865 30842707 PMC6392602

[25] Danielian M, Danielian A, Harutunyan L, Ishiyama A, Akaragian S. Parental perspectives on the quality of life of children with cochlear implants in Armenia. IJQHC Communications. 2023;3(1). doi: 10.1093/ijcoms/lyad001

[26] Nunes T, Pretzlik U, Ilicak S. Validation of a parent outcome questionnaire from pediatric cochlear implantation. J Deaf Stud Deaf Educ. 2005;10(4):330–56. doi: 10.1093/deafed/eni027 15932922

[27] Zamanzadeh V, Ghahramanian A, Rassouli M, Abbaszadeh A, Alavi-Majd H, Nikanfar A-R. Design and implementation content validity study: Development of an instrument for measuring Patient-Centered Communication. J Caring Sci. 2015;4(2):165–78. doi: 10.15171/jcs.2015.017 26161370 PMC4484991

[28] Taber KS. The use of Cronbach’s alpha when developing and reporting research instruments in science education. Res Sci Educ. 2017;48(6):1273–96. doi: 10.1007/s11165-016-9602-2

[29] Bronfenbrenner U. The ecology of human development: Experiments by nature and design. Cambridge, MA: Harvard University Press. 1979.

