# Supplementary material for: Assessing the quality of life among children with cochlear implants in Saudi Arabia
Source: PLoS One. 2026 Feb 20;21(2):e0343237. doi: 10.1371/journal.pone.0343237 (PMC12923006; doi:10.1371/journal.pone.0343237)
Supplement: S1 Appendix — (PDF) [file pone.0343237.s001.pdf]

Appendix A

Closed Format Questionnaire

Thinking about your child now, compared to before implantation...	Strongly Agree	Agree	Neither agree or disagree	Disagree	Strongly disagree	
Communication						
Communication is difficult even with people he knows well	❑	❑	❑	❑	❑	c1
The quality of his speech gives me cause for concern	❑	❑	❑	❑	❑	c2
His use of spoken language has developed greatly	❑	❑	❑	❑	❑	c3
He communicates easily and effectively using his implant	❑	❑	❑	❑	❑	c4
Before implantation he obtained no benefit at all from his hearing aids	❑	❑	❑	❑	❑	c5
We can now chat even when he cannot see my face (eg in the car or in the dark)	❑	❑	❑	❑	❑	c6
A major improvement has been in his communication ability	❑	❑	❑	❑	❑	c7
General functioning						
He is totally reliant on his implant all the time	❑	❑	❑	❑	❑	g1
I can now let him play outside as he is aware of the sound of traffic	❑	❑	❑	❑	❑	g2
He knows when I want his attention because he can hear me call	❑	❑	❑	❑	❑	g3
He is more aware of things going on around him	❑	❑	❑	❑	❑	g4
He is still unable to cope in new situations	❑	❑	❑	❑	❑	g5
He can now amuse himself listening to music or watching TV or playing games	❑	❑	❑	❑	❑	g6
He now listens to the radio when he is on his own	❑	❑	❑	❑	❑	g7
Self-reliance						
A significant change has been improvement in confidence	❑	❑	❑	❑	❑	r1
He is insecure lacking confidence and assertiveness	❑	❑	❑	❑	❑	r2
He was very dependent on us before implantation	❑	❑	❑	❑	❑	r3
I can seldom leave him to do something on his own	❑	❑	❑	❑	❑	r4
He is as independent as most other children of his age	❑	❑	❑	❑	❑	r5
Well-being and happiness						
He continues to be a happy child and good fun to be with	❑	❑	❑	❑	❑	h1
He is happier now than before he had his implant	❑	❑	❑	❑	❑	h2
He is less frustrated than before he had the implant	❑	❑	❑	❑	❑	h3
His behaviour has improved since he had his implant	❑	❑	❑	❑	❑	h4
He still shows signs of frustration in his behaviour	❑	❑	❑	❑	❑	h5
He has become awkward and uncooperative since getting his implant	❑	❑	❑	❑	❑	h6
Before implantation he was quiet and reserved	❑	❑	❑	❑	❑	h7
He has become argumentative since getting his implant	❑	❑	❑	❑	❑	h8
Social relationships	❑	❑	❑	❑	❑	
He was socially isolated before getting his implant	❑	❑	❑	❑	❑	s1
Easier communication has led to improved family relationships	❑	❑	❑	❑	❑	s2
He is sociable now and makes friends easily	❑	❑	❑	❑	❑	s3
Now he is talkative and engages others in conversation	❑	❑	❑	❑	❑	s4
He is sociable within the family	❑	❑	❑	❑	❑	s5
He does not make friends easily outside the family	❑	❑	❑	❑	❑	s6
He shares in family situations more than before implantation	❑	❑	❑	❑	❑	s7
He does not have a close relationship with his grandparents	❑	❑	❑	❑	❑	s8
He takes part in family relationships on an equal footing with other members	❑	❑	❑	❑	❑	s9
In new situations he remains isolated and finds it difficult to make friends	❑	❑	❑	❑	❑	s10
His relationship with brothers and sisters has improved	❑	❑	❑	❑	❑	s11
He relies on a member of the family for all his social interactions	❑	❑	❑	❑	❑	s12
There has been a general improvement in family relationships since implantation	❑	❑	❑	❑	❑	s13

	Strongly Agree	Agree	Neither agree or disagree	Disagree	Strongly disagree	
Thinking now about your child's education ...						
Education						
He is keeping up well with children of his own age at school	❑	❑	❑	❑	❑	e1
He is unable to cope with mainstream schooling	❑	❑	❑	❑	❑	e2
He is totally reliant on his implant at school	❑	❑	❑	❑	❑	e3
The local school and support services adequately meet all our needs concerning the use of his implant at school	❑	❑	❑	❑	❑	e4
I am not happy with his educational placement at present	❑	❑	❑	❑	❑	e5
Continuing links between the Implant Centre and his school are essential	❑	❑	❑	❑	❑	e6
His local teacher needs specialist advice, reassurance and help from the Implant Centre to set appropriate expectations	❑	❑	❑	❑	❑	e7
Parents should have a choice in the use of sign language at school	❑	❑	❑	❑	❑	e8
We feel the need for advice from the Implant Centre concerning his future	❑	❑	❑	❑	❑	e9
We are reliant on the Implant Centre for technical advice about his implant	❑	❑	❑	❑	❑	e10
I am concerned about his future school placement	❑	❑	❑	❑	❑	e11
Thinking now about your experiences of the process of implantation ...						
Process of implantation						
The whole process of implantation was intrusive	❑	❑	❑	❑	❑	p1
It has been a problem getting someone to look after the family when we go to the Implant Centre	❑	❑	❑	❑	❑	p2
Other children in the family resented the time and attention taken up by the implant	❑	❑	❑	❑	❑	p3
Travelling to the Implant Centre is a burden on our time	❑	❑	❑	❑	❑	p4
It has been hard to take time off work for the appointments at the Implant Centre	❑	❑	❑	❑	❑	p5
The process of implantation was no more intrusive than expected	❑	❑	❑	❑	❑	p6
The costs of travel to the Implant Centre are a problem	❑	❑	❑	❑	❑	p7
It is essential that he is encouraged to wear the processor all the time	❑	❑	❑	❑	❑	p8
Only experienced teams should carry out cochlear implantation	❑	❑	❑	❑	❑	p9
It is important to encourage children to listen through their implants athome	❑	❑	❑	❑	❑	p10
A positive attitude is a great help towards successful use of the implant	❑	❑	❑	❑	❑	p11
Regular tuning and checking of the implant system are essential	❑	❑	❑	❑	❑	p12
Feedback from assessments at the Implant Centre is very useful	❑	❑	❑	❑	❑	p13
The most important factor in choosing an implant device is its reliability	❑	❑	❑	❑	❑	p14
At least one visit per year by Implant Centre staff to home/school is essential	❑	❑	❑	❑	❑	p15
I wish to participate in meetings with other families having an implanted child	❑	❑	❑	❑	❑	p16
There is a need for lifetime support from the Implant Centre	❑	❑	❑	❑	❑	p17
It is important for Implant Centre staff regularly to observe his use of the implant at school/home	❑	❑	❑	❑	❑	p18
Before proceeding with implantation, parents should obtain as much information and advice as possible	❑	❑	❑	❑	❑	p19
The programme at the Implant Centre should emphasise speaking and listening	❑	❑	❑	❑	❑	p20
Signing support is helpful for a considerable time after implantation	❑	❑	❑	❑	❑	p21
It was useful to meet another family with an implanted child before deciding on an implant	❑	❑	❑	❑	❑	p22
Speech and language therapy is important after implantation	❑	❑	❑	❑	❑	p23

	Strongly Agree	Agree	Neither agree or disagree	Disagree	Strongly disagree	
Thinking now about the effects of implantation on carers...						
Effects of implantation						
I worry that the implant will break down	❑	❑	❑	❑	❑	i1
Immediately after implantation his behaviour deteriorated	❑	❑	❑	❑	❑	i2
I am confident that the Implant Centre will sort out any problems that occur	❑	❑	❑	❑	❑	i3
The whole process of implantation is still stressful	❑	❑	❑	❑	❑	i4
I am happy about his progress at school	❑	❑	❑	❑	❑	i5
I am worried that his speech will not be clear enough for him to be understood by strangers	❑	❑	❑	❑	❑	i6
I am confident that long term electrical stimulation will not be a problem	❑	❑	❑	❑	❑	i7
Immediately after implantation his ability to communicate was poorer	❑	❑	❑	❑	❑	i8
Progress during the first few months seemed very slow	❑	❑	❑	❑	❑	i9
Thinking about making the decision to go ahead with implantation...						
Decision to implant						
Making the decision to proceed with implantation was the most difficult part for me	❑	❑	❑	❑	❑	d1
Wondering whether I had made the right decision was a worry during the first few months after implantation	❑	❑	❑	❑	❑	d2
It was a difficult time waiting for the results of the assessments before implantation	❑	❑	❑	❑	❑	d3
It was a relief seeing him respond to sound for the first time	❑	❑	❑	❑	❑	d4
I chose implantation for my child so he would have a chance to become part of the hearing world	❑	❑	❑	❑	❑	d5
I expected him to learn to talk once he had his implant	❑	❑	❑	❑	❑	d6
I hoped the implant would ultimately give him better job prospects	❑	❑	❑	❑	❑	d7
Progress after implantation has exceeded my expectations	❑	❑	❑	❑	❑	d8
I worry that ultimately he may be neither part of the deaf nor the hearing world	❑	❑	❑	❑	❑	d9
I am concerned that my child will be rejected by the deaf community because of the implant	❑	❑	❑	❑	❑	d10
It was important to me that my child could hear sounds from traffic for safety reasons	❑	❑	❑	❑	❑	d11
I worry that he will blame me for my decision for him to have an implant	❑	❑	❑	❑	❑	d12
It all seems worthwhile when he tells me easily about what he has been doing	❑	❑	❑	❑	❑	d13
I am encouraged when I see him communicating easily with hearing friends	❑	❑	❑	❑	❑	d14
I believe now that my child will have reasonable prospects for employment	❑	❑	❑	❑	❑	d15
Finally, thinking about what is involved caring for an implanted child ...						
Supporting the child						
He has needed more help from me since he received his implant	❑	❑	❑	❑	❑	u1
A parent of a child with an implant needs to be patient as benefits may take time to show	❑	❑	❑	❑	❑	u2
I find it easier to communicate with him by speaking than by signing	❑	❑	❑	❑	❑	u3
I give him the same amount of help as before his implant	❑	❑	❑	❑	❑	u4
The help I give him has become more productive now he has his implant	❑	❑	❑	❑	❑	u5
Parents who decide on implantation need to be prepared for a lot of hard work	❑	❑	❑	❑	❑	u6
A lot of help at first means a child needs less help later	❑	❑	❑	❑	❑	u7
I find it easier to communicate with him now he has an implant	❑	❑	❑	❑	❑	u8
I get more time to myself because of his increased independence	❑	❑	❑	❑	❑	u9

Comments

Thank you very much for taking the time to complete this questionnaire

جودة حياة أطفال زارعي القوقعة الإلكترونية

Quality of life in children with cochlear implants

فكر في طفلك الآن مقارنةً بما قبل الزراعة

محور التواصل

أعارض بشدة	أعارض	محايد	أو افق	أو افق بشدة	
❑	❑	❑	❑	❑	التواصل صعب حتى مع الأشخاص الذين يعرفهم جيدًا
❑	❑	❑	❑	❑	جودة كلامه تثير قلقي
❑	❑	❑	❑	❑	استخدامه للغة المنطوقة تطور بشكل كبير
❑	❑	❑	❑	❑	يتواصل بسهولة وفعالية بعد زراعة القوقعة
❑	❑	❑	❑	❑	قبل زراعة القوقعة، لم يكن يحصل على أي فائدة من السماعات
❑	❑	❑	❑	❑	يمكننا الآن التحدث حتى عندما لا يرى وجهي (مثلاً في السيارة أو في الظلام)
❑	❑	❑	❑	❑	تحسن كبير حدث في قدرته على التواصل بعد زراعة القوقعة

1. **الأداء العام**

أعارض بشدة	أعارض	لا أو افق ولا أعترض	أو افق	أو افق بشدة	
❑	❑	❑	❑	❑	يعتمد تمامًا على جهاز زراعة القوقعة طوال الوقت
❑	❑	❑	❑	❑	يمكنني الآن السماح له باللعب في الخارج لأنه يدرك الأصوات ويستجيب بشكل جيد في حال تم مناداته
❑	❑	❑	❑	❑	يعرف متى أريد انتباهه لأنه يسمعني أناديه
❑	❑	❑	❑	❑	إنه أكثر إدراكًا لما يحدث من حوله
❑	❑	❑	❑	❑	لا يزال غير قادر على التأقلم في المواقف الجديدة.
❑	❑	❑	❑	❑	يمكنه الآن تسلية نفسه بالاستماع إلى الأصوات المحببة لدية أو مشاهدة التلفاز أو اللعب
❑	❑	❑	❑	❑	هو الآن يستمع إلى الراديو او أي مادة صوتية عندما يكون بمفرده.

2. **الاعتماد على النفس**

أعارض بشدة	أعارض	لا أو افق ولا أعترض	أو افق	أو افق بشدة	
❑	❑	❑	❑	❑	تغيير كبير حدث في زيادة ثقته بنفسه
❑	❑	❑	❑	❑	هو غير واثق من نفسه ويفتقر إلى الثقة والحزم
❑	❑	❑	❑	❑	كان معتمدًا جدًا علينا قبل زراعة القوقعة
❑	❑	❑	❑	❑	نادراً ما أستطيع تركه ليقوم بشيء بمفرده.
❑	❑	❑	❑	❑	هو مستقل مثل معظم الأطفال في عمره.

3. الرفاهية والسعادة

أعارض بشدة	أعارض	لا أو افق ولا أعترض	أو افق	أو افق بشدة	
❑	❑	❑	❑	❑	يظل بأنه طفلاً سعيدًا وممتعًا مع والديه
❑	❑	❑	❑	❑	هو أكثر سعادة الآن مما كان قبل زراعة القوقعة
❑	❑	❑	❑	❑	هو أقل إحباطًا مما كان قبل زراعة القوقعة
❑	❑	❑	❑	❑	تحسن سلوكه منذ حصوله على زراعة القوقعة
❑	❑	❑	❑	❑	لا يزال يظهر علامات الإحباط في سلوكه
❑	❑	❑	❑	❑	أصبح صعبًا وغير متعاون منذ حصوله على زراعة القوقعة
❑	❑	❑	❑	❑	قبل زراعة القوقعة كان هادئًا ومنطويًا
❑	❑	❑	❑	❑	أصبح مجادلًا منذ حصوله على قبل زراعة القوقعة

4. العلاقات الاجتماعية

أعارض بشدة	أعارض	لا أو افق ولا أعترض	أو افق	أو افق بشدة	
❑	❑	❑	❑	❑	كان معزولًا اجتماعيًا قبل حصوله على زراعة القوقعة
❑	❑	❑	❑	❑	التواصل السهل أدى إلى تحسين العلاقات الأسرية
❑	❑	❑	❑	❑	هو اجتماعي الآن ويُكون صداقات بسهولة
❑	❑	❑	❑	❑	الآن هو ثرثار ويشارك الآخرين في الحديث
❑	❑	❑	❑	❑	هو اجتماعي داخل الأسرة
❑	❑	❑	❑	❑	لا يُكون صداقات بسهولة خارج الأسرة
❑	❑	❑	❑	❑	يشارك في المواقف الأسرية أكثر مما كان قبل زراعة القوقعة
❑	❑	❑	❑	❑	لا تربطه علاقة وثيقة مع أجداده
❑	❑	❑	❑	❑	يشارك في العلاقات الأسرية على قدم المساواة مع الآخرين

أعارض بشدة	أعارض	لا أو افق ولا أعترض	أو افق	أو افق بشدة	
❑	❑	❑	❑	❑	في المواقف الجديدة يظل معزولًا ويجد صعوبة في تكوين صداقات
❑	❑	❑	❑	❑	تحسنت علاقته مع إخوته وأخواته
❑	❑	❑	❑	❑	يعتمد على أحد أفراد الأسرة في جميع تفاعلاته الاجتماعية
❑	❑	❑	❑	❑	حدث تحسن عام في العلاقات الأسرية منذ حصوله على زراعة القوقعة

5. التعليم

فكر الآن في تعليم طفلك...

أعارض بشدة	أعارض	لا أو افق ولا أعترض	أو افق	أو افق بشدة	
❑	❑	❑	❑	❑	هو يتماشى جيدًا مع الأطفال في سنه في المدرسة
❑	❑	❑	❑	❑	لا يستطيع التعامل مع التعليم العادي
❑	❑	❑	❑	❑	يعتمد تمامًا على زرعة القوقعة في المدرسة
❑	❑	❑	❑	❑	المدرسة وخدمات الدعم تلبي بشكل كافٍ جميع احتياجاتنا المتعلقة باستخدام زرعته في المدرسة.
❑	❑	❑	❑	❑	أنا غير راضٍ عن مكانه التعليمي الحالي
❑	❑	❑	❑	❑	استمرار التواصل / التفاهم بين مركز زراعة القوقعة المدرسة أمر ضروري.
❑	❑	❑	❑	❑	يحتاج معلمه في المدرسة إلى نصائح متخصصة ودعم ومساعدة من مركز زراعة القوقعة لوضع التوقعات المناسبة حول الطالب
❑	❑	❑	❑	❑	يجب أن يكون للأهالي خيار استخدام لغة الإشارة في المدرسة
❑	❑	❑	❑	❑	نشعر بالحاجة إلى نصيحة من مركز زراعة القوقعة بالمستشفى بشأن مستقبله
❑	❑	❑	❑	❑	نعتمد على مركز زراعة القوقعة في المستشفى للحصول على نصائح تقنية حول زراعة القوقعة
❑	❑	❑	❑	❑	أنا قلق بشأن مكانه التعليمي المستقبلي

6. عملية الزراعة

فكر الآن في تجربتك مع عملية الزراعة...

أعارض بشدة	أعارض	لا أو افق ولا أعترض	أو افق	أو افق بشدة	
❑	❑	❑	❑	❑	كانت عملية زراعة القوقعة بأكملها مزعجة وصعبة
❑	❑	❑	❑	❑	كان من الصعب الحصول على شخص يعتني بالأسرة عندما نذهب إلى مركز زراعة القوقعة
❑	❑	❑	❑	❑	الأطفال الآخرون في العائلة استاءوا من الوقت الذي أخذته عملية زراعة القوقعة
❑	❑	❑	❑	❑	السفر إلى مركز زراعة القوقعة (المستشفى) يشكل عبئًا على وقتنا
❑	❑	❑	❑	❑	كان من الصعب أخذ إجازة من العمل لمواعيد مركز زراعة القوقعة (المستشفى)
❑	❑	❑	❑	❑	كانت عملية زراعة القوقعة أقل تعقيداً مما كنت أتوقع
❑	❑	❑	❑	❑	تكاليف السفر إلى مركز زراعة القوقعة (المستشفى) يشكل عبئًا
❑	❑	❑	❑	❑	من الضروري أن يتم تشجيعه على ارتداء المعالج طوال الوقت
❑	❑	❑	❑	❑	يجب أن تقوم فرق ذات خبرة فقط بإجراء زرع القوقعة
❑	❑	❑	❑	❑	من المهم تشجيع الأطفال على الاستماع باستخدام زارعاتهم السمعية في المنزل.
❑	❑	❑	❑	❑	الدعم الإيجابي يعد عاملاً مساعداً كبيراً نجاح زراعة القوقعة
❑	❑	❑	❑	❑	الصيانة الدورية وفحص نظام زراعة القوقعة أمران أساسيان.
❑	❑	❑	❑	❑	التغذية الراجعة من مركز زراعة القوقعة (المستشفى) مفيدة جدًا
❑	❑	❑	❑	❑	العامل الأهم في اختيار جهاز زراعة القوقعة هو موثوقيته
❑	❑	❑	❑	❑	يجب أن يكون هناك زيارة واحدة على الأقل في السنة من موظفي مركز زراعة القوقعة (المستشفى) إلى المنزل/المدرسة
❑	❑	❑	❑	❑	أرغب في المشاركة في اجتماعات مع عائلات أخرى لديها طفل زارع قوقعة
❑	❑	❑	❑	❑	هناك حاجة لدعم مستمر مدى الحياة من مركز زراعة القوقعة (المستشفى)
❑	❑	❑	❑	❑	من المهم أن يقوم موظفو مركز زراعة القوقعة (المستشفى) بمراقبة استخدامه لزراعة القوقعة في المدرسة والمنزل بانتظام.
❑	❑	❑	❑	❑	قبل المضي قدمًا في زراعة القوقعة، يجب على الأهل الحصول على أكبر قدر ممكن من المعلومات والنصائح
❑	❑	❑	❑	❑	يجب أن يركز البرنامج في زراعة القوقعة (المستشفى) على مهارات التحدث والاستماع.
❑	❑	❑	❑	❑	الدعم بلغة الإشارة يكون مفيدًا لفترة معتبرة بعد عملية زراعة القوقعة
❑	❑	❑	❑	❑	كان من المفيد مقابلة عائلة أخرى لديها طفل زارع قوقعة قبل اتخاذ قرار زراعة القوقعة

	أعارض بشدة	أعارض	لا أو افق ولا أعترض	أو افق	أو افق بشدة
معالجة الكلام واللغة ضروري بعد عملية زراعة القوقعة	❑	❑	❑	❑	❑

7. **تأثير الزرع**

فكر الآن في تأثير الزرع على مقدمي الرعاية...

	أعارض بشدة	أعارض	لا أو افق ولا أعترض	أو افق	أو افق بشدة
قلق من أن يتعطل الجهاز المزروع.	❑	❑	❑	❑	❑
تدهور سلوكه فورًا بعد عملية زراعة القوقعة.	❑	❑	❑	❑	❑
أنا واثق من أن مركز زراعة القوقعة (المستشفى) سيحل أي مشاكل تحدث	❑	❑	❑	❑	❑
عملية زراعة القوقعة بأكملها لا تزال مرهقة	❑	❑	❑	❑	❑
أنا سعيد بتقدمه التعليمي في المدرسة	❑	❑	❑	❑	❑
أقلق من أن كلامه لن يكون واضحًا بما يكفي لفهمه من قبل الأشخاص الذين لا يعرفهم	❑	❑	❑	❑	❑
أنا واثق من أن التحفيز الكهربائي طويل الأمد لن يكون مشكلة	❑	❑	❑	❑	❑
بعد زراعة القوقعة مباشرةً، كانت قدرته على التواصل أقل	❑	❑	❑	❑	❑
التقدم بعد عملية زراعة القوقعة خلال الأشهر القليلة الأولى بدا بطيئًا للغاية	❑	❑	❑	❑	❑

8. **قرار الزرع**

فكر الآن في اتخاذ قرار المضي قدمًا في زراعة القوقعة..

	أعارض بشدة	أعارض	لا أو افق ولا أعترض	أو افق	أو افق بشدة
كان اتخاذ قرار المضي قدمًا في عملية زراعة القوقعة هو الجزء الأصعب بالنسبة لي.	❑	❑	❑	❑	❑
كان القلق بشأن ما إذا كنت قد اتخذت القرار الصحيح يشغلني خلال الأشهر القليلة الأولى بعد عملية زراعة القوقعة.	❑	❑	❑	❑	❑
كانت فترة انتظار نتائج التقييمات قبل عملية زراعة القوقعة وقتًا صعبًا.	❑	❑	❑	❑	❑
كانت لحظة رائعة عند رؤيته وهو يستجيب للأصوات لأول مرة.	❑	❑	❑	❑	❑
كان قرار زراعة القوقعة لطفلي ليندمج مع مجتمع السامعين	❑	❑	❑	❑	❑

أعارض بشدة	أعارض	لا أو افق ولا أعترض	أو افق	أو افق بشدة	
❑	❑	❑	❑	❑	كانت التوقعات انه سوف يتعلم الكلام بمجرد حصوله على زراعة القوقعة
❑	❑	❑	❑	❑	كنت آمل أن توفر له زراعة القوقعة في نهاية المطاف فرص عمل أفضل مستقبلاً.
❑	❑	❑	❑	❑	التقدم الذي حصل عليه طفلي بعد عملية زراعة القوقعة قد تجاوز توقعاتي.
❑	❑	❑	❑	❑	أقلق من أنه في النهاية قد لا يكون جزءًا من مجتمع الصم أو مجتمع السامعين.
❑	❑	❑	❑	❑	أشعر بالقلق من أن يتم رفض طفلي من قبل مجتمع الصم بسبب زراعة القوقعة
❑	❑	❑	❑	❑	كان من المهم بالنسبة لي أن يتمكن طفلي من سماع أصوات حركة المرور لأسباب تتعلق بالسلامة.
❑	❑	❑	❑	❑	أخشى أن يلومني على قراري لعمل عملية زراعة القوقعة
❑	❑	❑	❑	❑	يبدو أن كل ذلك يستحق العناء عندما يخبرني بكل سهولة عما كان يفعله.
❑	❑	❑	❑	❑	أشعر بالتشجيع عندما أراه يتواصل بسهولة مع أصدقائه من السامعين.
❑	❑	❑	❑	❑	أعتقد الآن أن طفلي سيكون لديه فرص معقولة للتوظيف

9. دعم الطفل

فكر الآن فيما يتطلبه رعاية طفل زارع قوقعة

أعارض بشدة	أعارض	لا أو افق ولا أعترض	أو افق	أو افق بشدة	يحتاج إلى المزيد من المساعدة مني منذ أن حصل على زراعة القوقعة.
❑	❑	❑	❑	❑	يجب على والد الطفل الذي يمتلك زراعة قوقعة أن يكون صبورًا لأن الفوائد قد تستغرق وقتًا لتظهر.
❑	❑	❑	❑	❑	أجد أنه من الأسهل التواصل معه عن طريق التحدث بدلاً من استخدام لغة الإشارة.
❑	❑	❑	❑	❑	أقدم له نفس القدر من المساعدة كما كنت أفعل قبل حصوله على زراعة القوقعة.
❑	❑	❑	❑	❑	المساعدة التي أقدمها له أصبحت أكثر إنتاجية الآن بعد حصوله على زراعة القوقعة
❑	❑	❑	❑	❑	يجب على الآباء الذين يقررون إجراء زراعة القوقعة أن يكونوا مستعدين للكثير من العمل الشاق.
❑	❑	❑	❑	❑	الكثير من المساعدة في البداية يعني أن الطفل سيحتاج إلى مساعدة أقل لاحقًا.
❑	❑	❑	❑	❑	أجد أنه من الأسهل التواصل معه الآن بعد زراعة القوقعة
❑	❑	❑	❑	❑	أحصل على وقت أطول لنفسي بسبب زيادة استقلاليته.

التعليقات

شكرًا جزيلاً على تخصيص وقتك لاستكمال هذا الاستبيان.
